# Spatial light-modulated stimulated Raman scattering (SLM-SRS) microscopy for rapid multiplexed vibrational imaging

**DOI:** 10.7150/thno.38551

**Published:** 2020-01-01

**Authors:** Kideog Bae, Wei Zheng, Zhiwei Huang

**Affiliations:** Optical Bioimaging Laboratory, Department of Biomedical Engineering, Faculty of Engineering, National University of Singapore, Singapore 117576.

**Keywords:** stimulated Raman scattering microscopy, spatial-light modulator, vibrational imaging

## Abstract

High speed imaging is pre-requisite for monitoring of dynamic processes in biological events. Here we report the development of a unique spatial light-modulated stimulated Raman scattering (SLM-SRS) microscopy that tailors the broadband excitation beam with sparse-sampling masks designed for rapid multiplexed vibrational imaging to monitor real-time cancer treatment effects and *in vivo* transport of drug solvent.

**Methods:** We design an optimal mask pattern that enables selection of predominant windows in SRS spectrum for collective excitation at the highest possible peak power, thus providing an improved signal-to-noise ratio (SNR) without compromise of chemical specificity. The mask pattern generated is applied to the broad excitation beam using a flexible spatial light modulator. The SLM module further offers complementary function whereby rapid scanning of SRS spectrum can be facilitated prior to the mask generation, thereby making the SLM-SRS system a stand-alone imaging platform.

**Results:** We demonstrate that SLM-SRS microscopy permits rapid multiplexed SRS imaging of polystyrene and polymethyl methacrylate beads in Brownian motion in dimethyl sulfoxide (DMSO) at 70 ms intervals without motion artiacts. We further apply SLM-SRS to monitor the therapeautic effect of mild alkaline solution on cancer cells, which shows immediate apoptotic response. Finally, we visualize *in vivo* penetration of DMSO into the plant tissue and evaluate acute toxicity of DMSO on cellulose and proteins within the tissue.

**Conclusion:** We develop novel SLM-SRS microscopy and affirm its broad applicability for rapid monitoring of dynamic biological processes at the subcellular and molecular level.

## Introduction

Stimulated Raman scattering (SRS) microscopy is a label-free vibrational imaging technique with high biochemical selectivity 1. When the two laser beams (termed as pump, ω_p_, and Stokes, ω_s_) coherently excite the specific molecular vibration of the sample under frequency-matching condition (i.e., ω_p_-ω_s_ = vibrational frequency of target molecules), SRS signal of up to 10^4^-fold higher than spontaneous Raman signal can be generated from the target molecules for high-speed vibrational imaging 1,2. Essentially, SRS signal is a portion of pump photons converted to the Stokes photons (termed the loss of pump intensity as stimulated Raman (SRL), or the gain of Stokes intensity as stimulated Raman gain (SRG)). Thus, the detection of SRL or SRG requires the use of amplitude-modulator and lock-in amplifier to demodulate the SRS signal from the excitation beams. With the high chemical specificity and sensitivity, SRS microscopy has been used for a wide range of biological and biomedical applications 3-6.

For observation of dynamic processes in living systems, high speed imaging is crucial to catering to the temporal resolution of the dynamic events. A simple strategy is to excite a single vibrational band in the Raman spectrum of each target component that offers the highest contrast against the interfering counterparts. The minimum spectral scanning steps used greatly shorten the overall data acquisition time, allowing rapid imaging of the dynamic processes such as protein metabolism in the mouse brain tissue and rapid uptake of antibiotics into biofilms 7,8. Given the narrow Raman linewidth of 10 to 15 cm^-1^, the technique is usually implemented with a picosecond (2-6 ps) laser source, whereby the signal-to-noise ratio (SNR) is capped by the relatively lower peak power compared to the shorter pulse lasers. Conversely, a higher signal level can be enjoyed with the use of femtosecond (<100 fs) laser beam, but the broad range of the resonance frequencies (>200 cm^-1^) would indiscriminately excite the multiple components in the sample, resulting in the deterioration of chemical selectivity. On the other hand, a spectroscopic detection method may rely on a programmable optical mask that tailors the broadband emission of Raman scattering 9. With the known Raman spectra of the sample as an input, the binary mask could be designed to selectively collect the vibrational frequencies of the target component that contribute to the higher sensitivity against the interfering counterparts. The multiplexed detection of the predominant frequencies further improves the SNR of Raman signal through Felgett's signal-to-noise advantage 10. The pulse-shaping is usually carried out using devices such as digital micromirror device (DMD) and spatial light modulator (SLM). Thus, post-processing is not required, making the sparse-sampling method suitable for rapid Raman imaging and SRS spectroscopy 11-13.

Here we report the development of a unique spatial light-modulated stimulated Raman scattering (SLM-SRS) microscopy which tailors the broadband excitation beam for multiplexed vibrational imaging with higher sensitivity. The pulse-shaping is achieved by using a sparse-sampling mask designed that allows collective excitation of the predominant spectral windows against the overlapping spectra. Due to the inverse time-frequency relationship between the time and spectral domains, the modulated beam with selective frequency components can attain higher peak power to improve signal-to-noise ratio without compromise of chemical specificity. For implementation, we incorporate a pulse-shaping module comprising a spatial light modulator (SLM) with flexible array design which offers dual functions: i) spectral patterning based on the sparse-sampling mask, and ii) rapid acquisition of SRS spectrum as prior knowledge of the sample needed for pattern generation. Such versatility makes our SLM-SRS imaging system stand-alone to target any unknown biological samples. With the rapid refresh rate offered by the SLM module, we successfully acquire multiplexed 4D SRS images of the functional responses of live cells to the alkaline environment as well as monitor *in vivo* penetration of dimethyl sulfoxide (DMSO) into a plant root for cytotoxicity assessment.

## Materials and Methods

### Implementation of SLM-SRS microscopy

**Figure 1:**
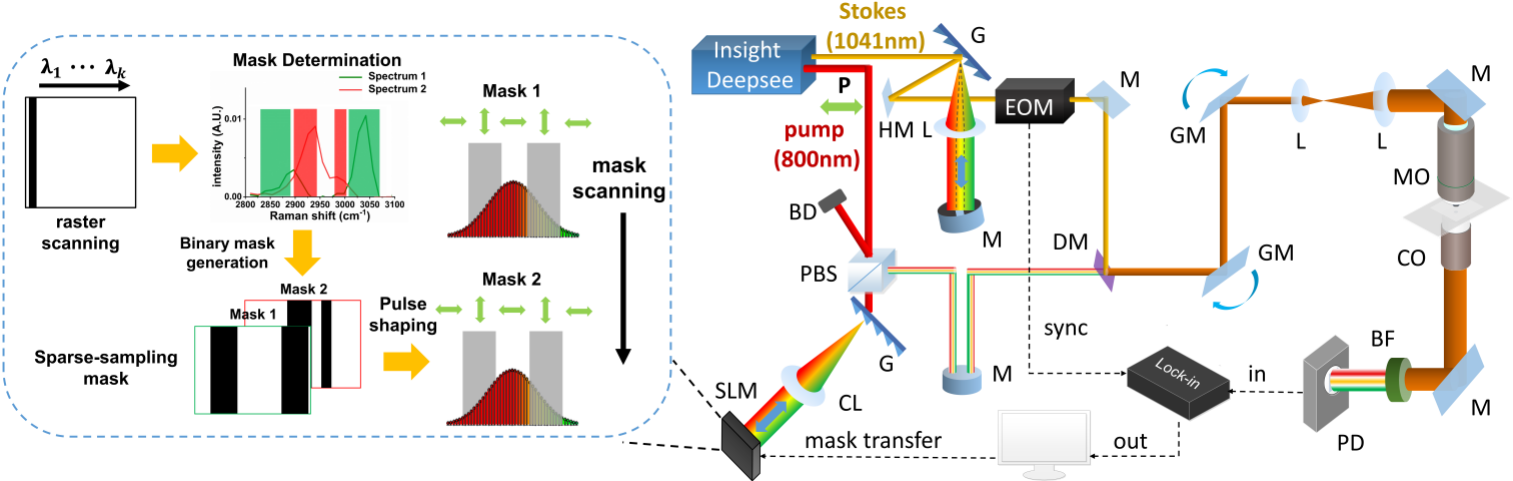
Schematic of SLM-SRS microscopy. M, mirror; HM, half mirror; G, grating; L, lens; CL, cylindrical lens; EOM, Electro-Optical Modulator; BD, beam dump; PBS, polarizing beamsplitter; P, polarization state; DM, dichroic mirror; GM; galvo mirror; MO, microscope objective; CO, condenser; BF, band-pass filter; PD, photodiode; SLM, spatial light modulator.

**Figure 2:**
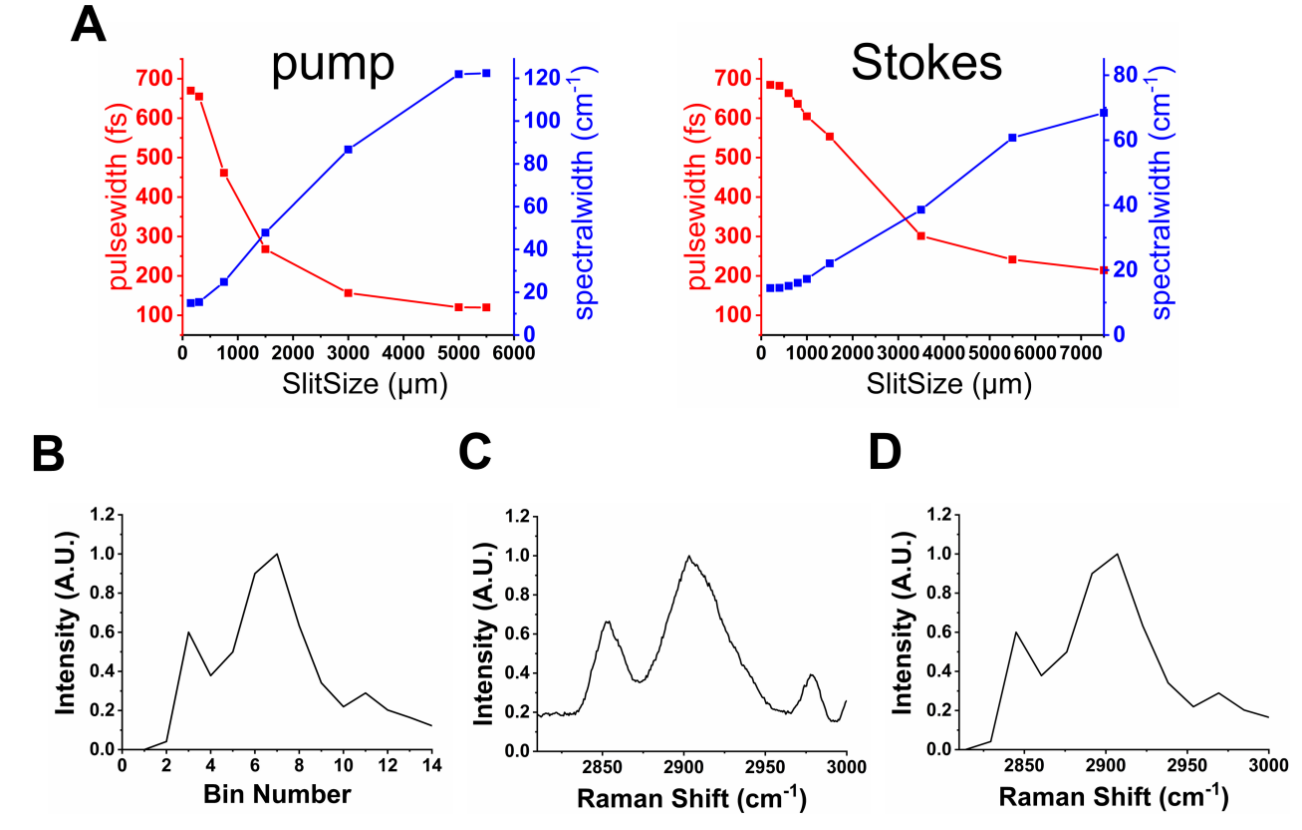
Characterization of SLM-SRS imaging system. (A) A plot of spectral resolution and pulsewidth against the slit size for the pump (left) and Stokes (right) beams. (B) A raw SRS spectrum of polystyrene (PS) bead by raster scanning of the SLM pixels. (C) Corresponding spontaneous Raman spectrum. (D) After comparison between (B) and (C), the bin numbers in (A) can be calibrated into Raman shifts.

The unique spatial light-modulated stimulated Raman scattering (SLM-SRS) microscopy system developed is depicted in Figure 1. Briefly, a 100-fs laser (InSight, Spectra Physics) at 80-MHz repetition rates gives dual outputs of a fixed 1041 nm beam (Stokes) and tunable (680-1300 nm) beam (pump). The Stokes beam is pulse-shaped to become narrow band using a unique folded 4-f system design. In our 4-f system, a reflection grating of 1200 grooves/mm and an achromatic lens with 200 mm focal length are used to form a dispersion line of ~ 8 mm. At the back focal plane of the lens, a slit of 400 µm is placed to cut off the spectrum of the Stokes beam to achieve spectral width of ~ 15 cm^-1^ (Figure 2A). Subsequently, the mirror behind the slit reflects the narrowed beam in the back-propagation direction. To make a spatial offset to separate the modulated beam from the incoming beam, a half mirror setting (KM100D, Thorlabs) is utilized to pass the reflected beam to the forward-propagation path. Afterwards, the amplitude of the beam is modulated at 20 MHz by an electro-optic modulator (EOM). Meanwhile, the pump beam is also passed through a separate folded 4-f system, which consists of a transmission grating (≈1400 grooves/mm, LightSmyth) and a cylindrical lens (f =150 mm, Newport), generating dispersion line of ~ 5 mm. The cylindrical lens is vertically placed so that the dispersion line is focused horizontally only in order to avoid photodamages to the SLM (HCA512-780-940, Meadowlark Optics, Inc.). The SLM selectively assigns the vertical polarization state to the desired spectral components (~15 cm^-1^; Figure 2A). The pulse-shaping is completed when the polarization beamsplitter at the end of the back-propagation path spatially separate them from the unmodulated spectral portion which remains as horizontally polarized. Thus, the modulated beam is directed to the forward-propagation path, whereas the unmodulated components are discarded. The two pulse-shaped beams are then combined at a dichroic mirror before laser-scanning microscope (MPM-4R, Thorlabs Inc.). The combined beams are focused onto the sample by a high-NA objective (CFI75 APOCHROMAT 25XW MP 1300, NA=1.1, Nikon.). After the SRS process in the sample, the excitation beams are collected by a condenser (CC Achromat/Aplanat, NA=1.4, Nikon) in the forward direction. In the detection path, the Stokes beam is spectrally removed by a bandpass filter (795/75, Semrock) so that the pump beam alone will be detected by the photodiode (FDS1010, Thorlabs Inc.). Finally, a lock-in amplifier is used to demodulate the stimulated Raman loss (SRL) from the pump beam for SRS imaging. We have measured the lateral and axial resolutions of the SLM-SRS system developed to be 0.45 and 2.1 µm, respectively, which are close to the theoretical values (Figure S1).

### Generation of sparse-sampling mask

In this work, we aim to design a unique binary mask pattern so that the intensity transmitted through the mask can represent a direct measurement of the dominant signals from a specific component against the other components. The working principle is as follows: Assuming that the sample consists of k number of chemical components, a_1_, a_2_, . . ., a_k_, and 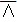
*_j_* stands for the stream of photons from specific chemical component, a_j_ (where 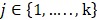). In reality, the detector measures the mixed photons from all a_j_ components in the sample. Thus, our goal is to unmix the detected photons and estimate all the individual 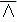
*_j_*
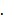
Given that each a*_j_* has its own spectra, S_ij_ (where 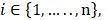
n = number of wavenumber channels in the spectroscopic system), the stream of mixed photons,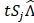, detected over time t is:


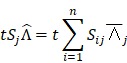
(1)

where 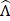 represents the rate of mixed photons experimentally detected by a detector.

To retrieve the unmixed photons, we could design a binary mask, M_ij_, that allows dominant photon generation from a_j_ over the other interfering components. Then, the measured signal (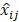) of the mixed photons from all components with the optical mask applied over T seconds can be modeled as:


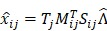
(2)

The mask can be optimized by minimizing the variance between 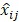 and the measured mean signal 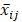
of specific photons from a_j_ as follows:


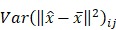
(3)

We can find an optimal mask (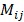) that makes 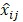
close to 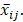 The details of the solution to the optimization problem are articulated in 9.

In consideration of the sparse-sampling mask design for multiplexed SRS imaging, the resonant frequencies are excited with a pulse laser whose energy density follows Gaussian distribution. Thus, the input spectra used for the mask design should reflect the intensity modulation caused by the excitation beam. The effective spectra (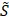) then can be taken in various forms depending on the peak position (peak wavelength) of the Gaussian excitation spectrum. We generate a set of sparse-sampling masks for each effective spectrum by scanning the peak wavelength (λ) of the excitation beam to find the optimal mask that gives the highest specificity for each component. At given effective spectra, we estimate the ratio (*ϕ_jλ_*) of spectral energy density between the target component and the sum of all other interfering components:


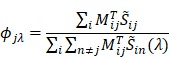
(4)

where n=n^th^ interfering component. Finally, we obtain a set of scores (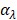) by summing over specificity values of all the components


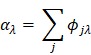
(5)

We then determine the mask 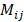 that gives the highest score to ensure both the highest possible SNR and overall chemical specificity for SRS imaging.

### Characterization of SLM-SRS imaging system

To construct sparse-sampling masks for an unknown sample, the prior knowledge of its individual spectra should be gained first. With the flexible array design of the SLM, the SLM-SRS system can generate the necessary SRS spectra by raster-scanning of the virtual 'slit' on the SLM (a vertical strip of pixels is assigned to 0 with the background pixels being 1 (inset in Figure 1)) prior to the SLM-SRS imaging (Figure 2B). The slit size is set at 300 µm to match the spectral resolution of ~15 cm^-1^ for the pump beam. With the energy density of the excitation beam following the Gaussian distribution, the intensity needs to be normalized by the intensity change in the two-photon absorption of Rhodamine-6G over the same scanning range 4. After calibration of the Raman shifts by comparing with the corresponding spontaneous Raman spectrum (Figure 2C), the SRS spectrum is finalized as shown in Figure 2D. In the case that the target components are spatially overlapped such that a mixed spectrum is obtained, the multivariate curve resolution (MCR) analysis may be applied to decompose them into individual components 14.

Based on the known spectra acquired, the sparse-sampling masks equal to the number of the major components identified can be generated. Given that the excitation beam is horizontally dispersed, the design of the sparse-sampling mask can be a series of vertical bins of binary values where the 'pass' region is assigned 0 (black) while the 'block' region 255 (white). The array values are then directly transferred to the pixel array on the SLM, each pixel of which rotates the polarization state of the incident beam from 0 to π/2 according to the pixel value (0 to 255). As a result, the light landing on the 'pass' region is assigned as vertical polarization so that it can be reflected by the subsequent polarization beamsplitter. The rest remains horizontally polarized for separation at the polarization beamsplitter, thereby achieving intensity modulation of the beam into desired pattern. The mask transfer is facilitated by the PCIe board, which enables rapid communication within 5 ms, allowing rapid multiplexed SRS imaging.

### Preparation of microsphere mixture

A mixture of 10 μm polystyrene (PS) and polymethyl methacrylate (PMMA) beads (Phosphorex) is prepared by the mixing microsphere solutions in water. 5 µl of solution is dropped on a coverslip and sealed for imaging.

### Preparation of biological samples

HeLa cells were cultured in Dulbecco's Modified Eagle Medium (DMEM) containing high glucose, glutamax, and 10% FBS. Cells were subcultured and plated on a bottom glass (MatTek Co., Ashland, MA) for one day in the incubator. Before imaging, the cells are washed with PBS twice to remove excess media. To create the alkaline environment at pH 8.5, 1 mL of NaHCO_3_ (0.5 M) is added to 3 mL of PBS media. Immediately, the 3D multiplexed SRS imaging at two channels (lipid and protein) is performed.

For *in vivo* plant imaging, *Arabidopsis thaliana* seedings were grown on petri dishes containing Murashige and Skoog medium (M5524, Sigma) under conditions (16 hr light/8 hr dark) in a growth cabinet. The temperature of the cabinet is set at 23 degrees in Celsius. The SRS experiments are carried out with 3-day-old seedlings.

## Results

**Figure 3:**
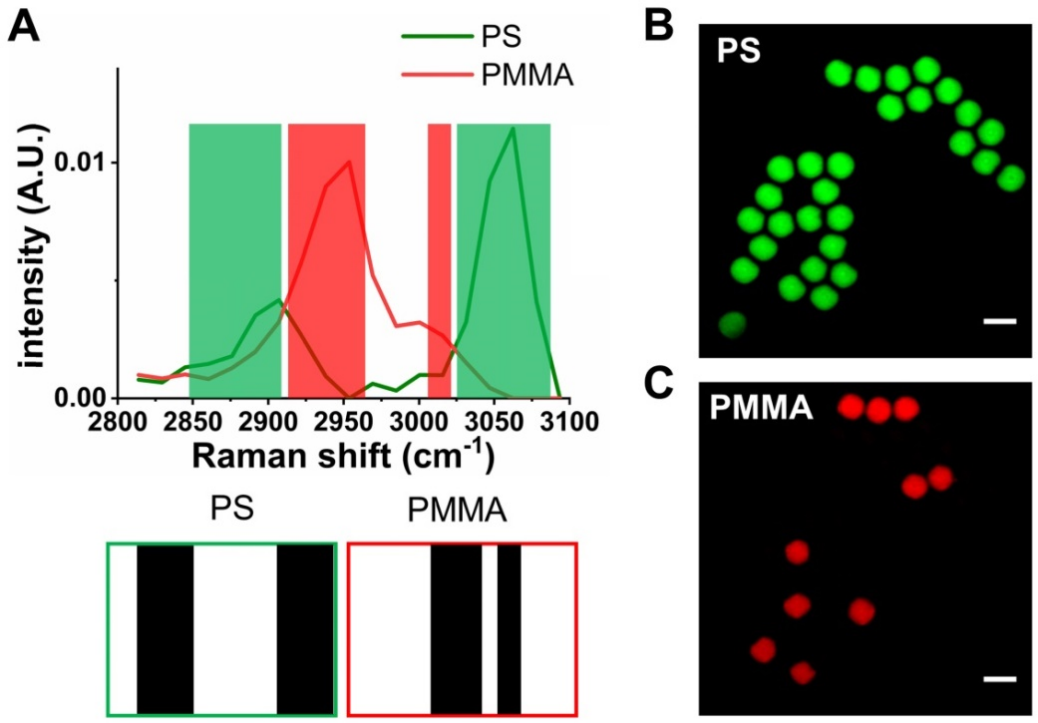
Validation of the SLM-SRS imaging technique. (A). SRS spectra of polystyrene (PS; green) and polymethyl methacrylate (PMMA; red). The shaded areas represent sparse-sampling masks with colors matching to each SRS spectra. The actual binary masks are displayed below: i.e., the black region has 0 value to allow transmission of the beam while the white region exhibits high optical attenuation. SLM-SRS images of (B) PS and (C) PMMA. All images are obtained within 1 s with 2.4 µs of pixel dwell time for 256 x 256 pixels (100 x 100 µm). 797 nm of the pump and 1041 nm of the Stokes beams are used. The average powers of the pump after passing through sparse-sampling mask for PS and PMMA are 6 and 5 mW, respectively. 20 mW of the Stokes beam is used throughout the experiment. Time constant of lock-in amplifier is set at 2 µs. Scale bar = 10 µm.

We firstly assess the performance of SLM-SRS microscopy with a mixture of 10 μm PS and PMMA beads prepared. Figure 3A shows the sparse-sampling masks for the two beads. In the overlay with their corresponding SRS spectra, we observe that the sparse-sampling algorithm only selects the spectral range where the target component exhibits a higher Raman cross-section than the interfering counterparts to avoid the compromise of chemical specificity. Further, the maximum coverage of the dominant range ensures the excitation of the sample with the highest possible peak power for achieving optimal SNR. Figure 3B-C shows the SLM-SRS images obtained with the two sparse-sampling masks, which depict clear separation between PS (green) and PMMA (red) beads.

**Figure 4:**
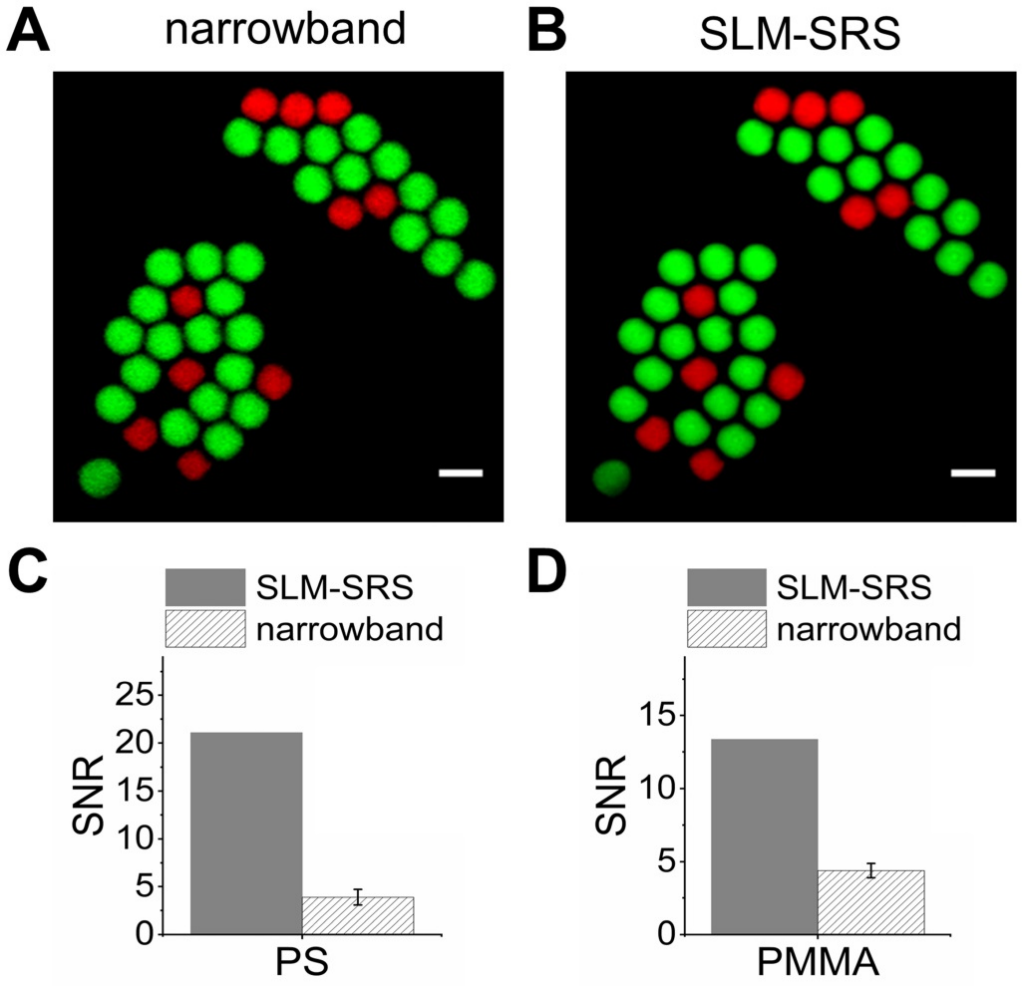
Comparison between SLM-SRS and narrowband SRS excitation. (A) Overlay of PS and PMMA images obtained at Raman shifts of 2950 and 3050 cm^-1^ using narrowband SRS excitation. (B) Overlay of Figure 3B-C. Measurements of signal-to-noise ratios for (C) PS beads and (D) PMMA beads in (A) and (B), respectively. The error bars are due to the repeated measurements of 10 PS/PMMA beads. The imaging conditions are the same as the parameters indicated in the figure legend of Figure 3.

We compare the SLM-SRS images with the counterparts obtained by raster-scanning using the SLM. Figure 4A shows the overlay of the most specific images of PS and PMMA beads selected from the hyperspectral SRS image stacks. Figure 4B shows the overlay of the previous SLM-SRS images (Figure 3B-C), illustrating a high specificity of SLM-SRS microscopy for PS and PMMA beams. Further, SLM-SRS gives a significantly higher SNR (5.4-fold for PS; 3-fold for PMMA; Figure 4C-D) than the narrowband excitation owing to its higher peak power at the same average power used for both excitation schemes.

**Figure 5:**
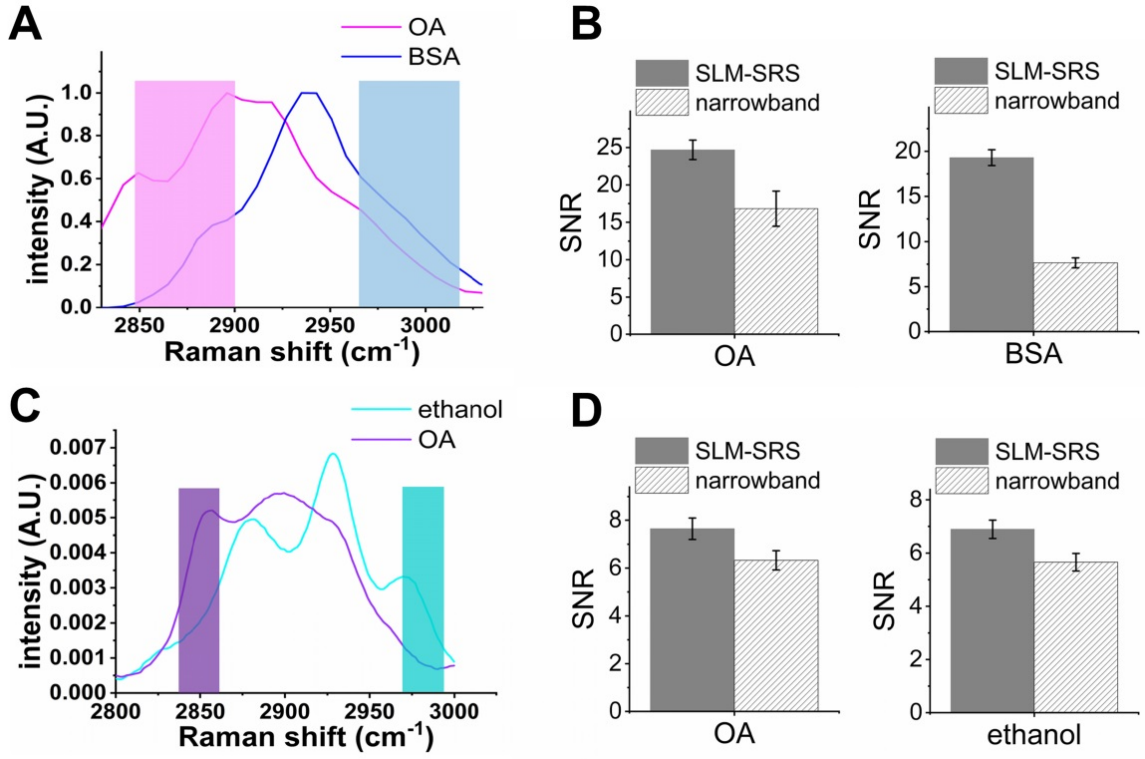
Evaluation on the performance of SLM-SRS microscopy with respect to the degree of spectral overlap. SRS spectra of (A) oleic acid (OA) and bovine serum albumin (BSA) and (C) OA and ethanol. The shaded areas represent sparse-sampling masks with colors matching to each SRS spectra. (B) and (D) comparison of the signal-to-noise ratio between SLM-SRS and narrowband excitations for chemical sets in (A) and (C), respectively.

Apart from the PS and PMMA beads mixture, we also evaluate the SNR improvement for other chemical mixtures with larger spectral overlap (Figure 5). In general, with the spectral overlap increasing, the excitation windows become narrower proportionally, resulting in a lower peak power employed for SRS generation. In the case of Figure 5B, we observe that the improvements in SNR compared to narrowband excitation are ~1.3 times for oleic acid (OA) and ~2.2 times for bovine serum albumin (BSA). In the extreme case (Figure 5C), the excitation window can be as narrow as the narrowband laser beam (~ 2 ps), resulting in an improvement factor of ~1. Therefore, the SNR enhancement of SLM-SRS microscopy developed can be maximized when the spectral overlap is moderate so that the size of the excitation windows generated lies in between the conventional narrowband (~20 cm^-1^) and broadband (~ 200 cm^-1^) excitation beams.

**Figure 6:**
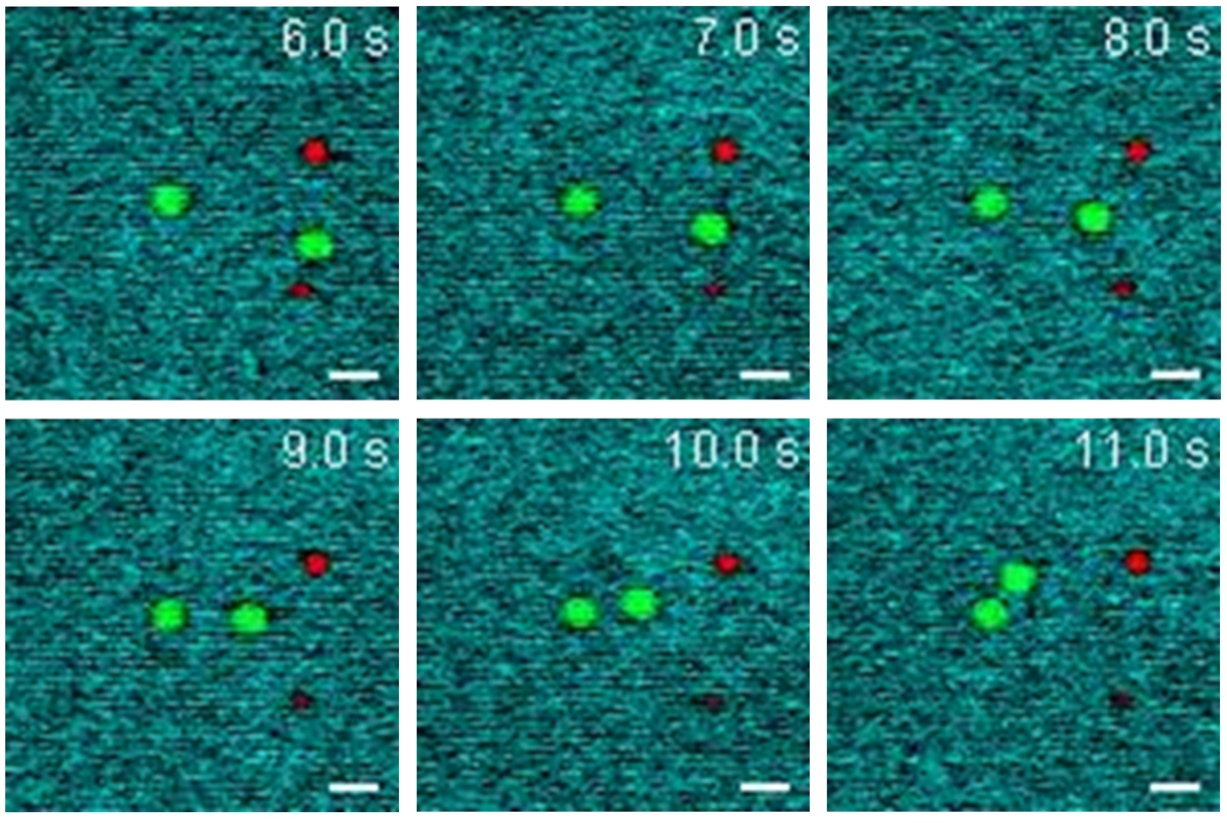
Dynamic SLM-SRS imaging of PS and PMMA beads with Brownian motion in DMSO solvent. The three-color images are acquired at intervals of 200 ms (Supplementary Movie S1) with 0.8 µs of pixel dwell time for the 40 × 40 µm (96 × 96 pixels). 797 nm of the pump and 1041 nm of the Stokes beams are used. The average powers of the pump after passing through spectral filters for PS, PMMA and DMSO are 10, 16 and 18 mW respectively. Time constant of lock-in amplifier is set at 0.8 µs. Scale bar = 5 µm.

To validate the rapid multiplexed imaging capability of SLM-SRS microscopy, we simultaneously visualize 4.5 μm PS and 3.0 μm PMMA beads with Brownian motions in DMSO. Prior to SLM-SRS imaging, we generate the sparse-sampling masks for the three chemicals and confirm their chemical specificity in the SRS image (see Supplementary Figure S2). Figure 6 shows the snapshots of three-color SRS images obtained by rapid mask scanning at the rate of 70 ms with SLM. In general, no smearing of the bead images due to motion artifacts is noticed, suggesting that the frame rate is sufficient to capture the dynamic motion of the beads in DMSO (For the full movie, see Movie S1).

**Figure 7:**
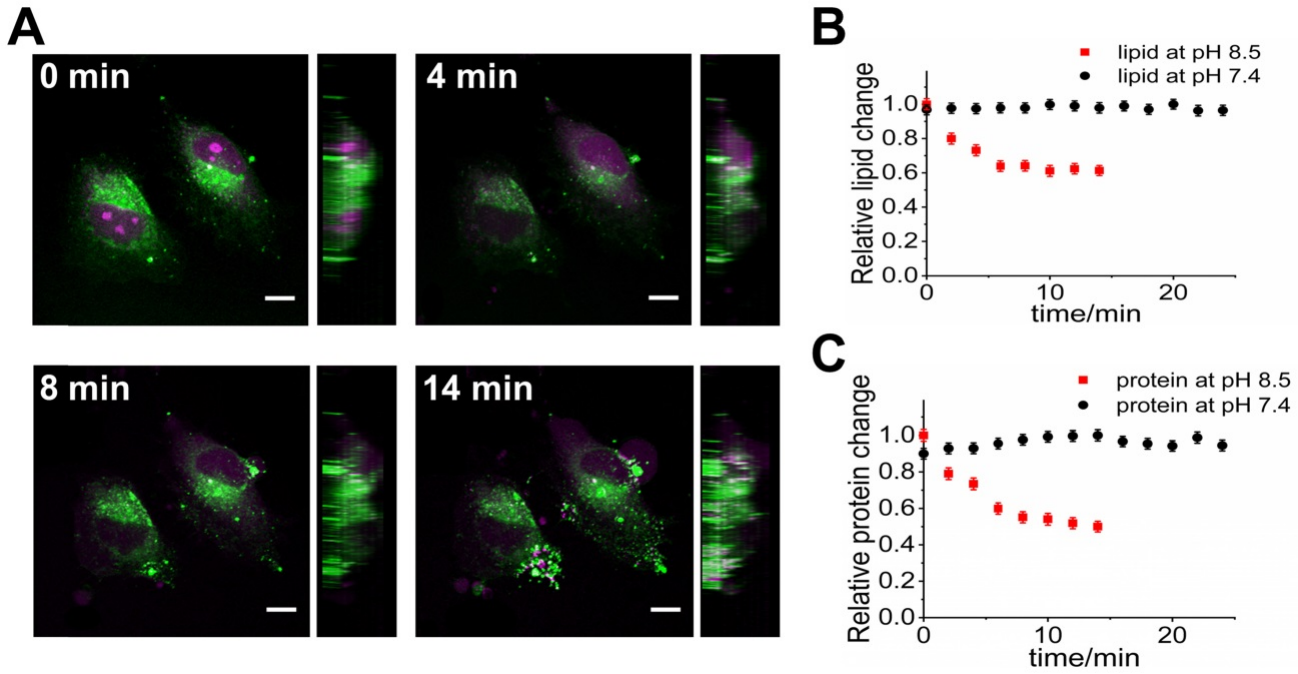
4D multiplexed SLM-SRS imaging of live HeLa cell at pH 8.5. (A) 3D visualization of HeLa cells at different times (0, 4, 8 and 14 min). For the full movie, see Movie S2. Side views are displayed on the right side. The image at the magenta channel is acquired with the sparse-sampling mask for the cellular protein and the green channel with another mask for the cellular lipid. Relative changes in the total contents of (B) lipids and (C) proteins within the cells over time at pH 8.5 (red dots) and at pH 7.4 (black dots; control). Number of cells involved for each measurement is 6 (i.e. n=6). All images are obtained within 3 s with 2.4 µs of pixel dwell time for 320 x 320 pixels (80 x 80 µm) and averaging 9 times. 799 nm of the pump and 1041 nm of the Stokes beams are used. The average powers of the pump after passing through sparse-sampling mask for cellular lipid and protein are 20 and 23 mW, respectively. 20 mW of the Stokes beam is used throughout the experiment. Time constant of lock-in amplifier is set at 2 µs. Scale bar = 7 µm.

We also demonstrate that SLM-SRS microscopy can be used to visualize dynamic processes in biological samples. In particular, we aim to observe the dynamic responses of the cancer cells to the alkaline environment which is known to exert deleterious effects on the tumor, thereby leading to therapeutic outcomes 15. Although its clinical effectiveness has been demonstrated, its mechanism at the cellular level remains elusive. Prior to a series of SRS imaging, we perform hyperspectral scanning on HeLa cells to obtain individual spectra of the major components in the cells. The resultant spectra after MCR decomposition are identified as cellular proteins and lipids 16 (Supplementary Figure S3). At pH 8.5 attained by adding sodium bicarbonate to the PBS buffer media, the 3D images of live HeLa cells are continuously scanned at a 2-min interval from t=0 to 14 min with alternating sparse-sampling masks for the cellular lipids and proteins. At both the cellular lipid and protein channels (Figure 7A), obvious signs of apoptosis can be observed. In the protein image, blebs are formed out of the cell body at 8 min onwards, indicating that cells are at the execution phase of apoptosis immediately after administration of sodium bicarbonate 17. The protein signals from the blebs are likely due to the presence of the actins and myosins originating from the cytoskeletons of the cells 18. Further, the degradation of chromatin within the nucleus is another characteristic of apoptosis highlighted in the protein channel. Given the direct linear relationship between the SRS voxel intensity and the concentration of resonant molecules, we measure the average voxel intensities of the cells at each channel to assess the relative lipid and protein changes during apoptosis. Figure 7B-C shows that both the cellular lipid and protein contents encounter a sharp decline in the first 10-min, reflecting the degradation of the biomolecules as an immediate deleterious effect on HeLa cells due to apoptosis 19. While in the control experiments where the live HeLa cells in PBS solution (pH 7.4) are monitored under the same imaging conditions, no significant changes in the concentration of biomolecules within the live cells are observed up to 25 min (Supplementary Movie. S3), confirming that the adverse phenomena observed in Fig. 7 are not caused by laser irradiation.

**Figure 8:**
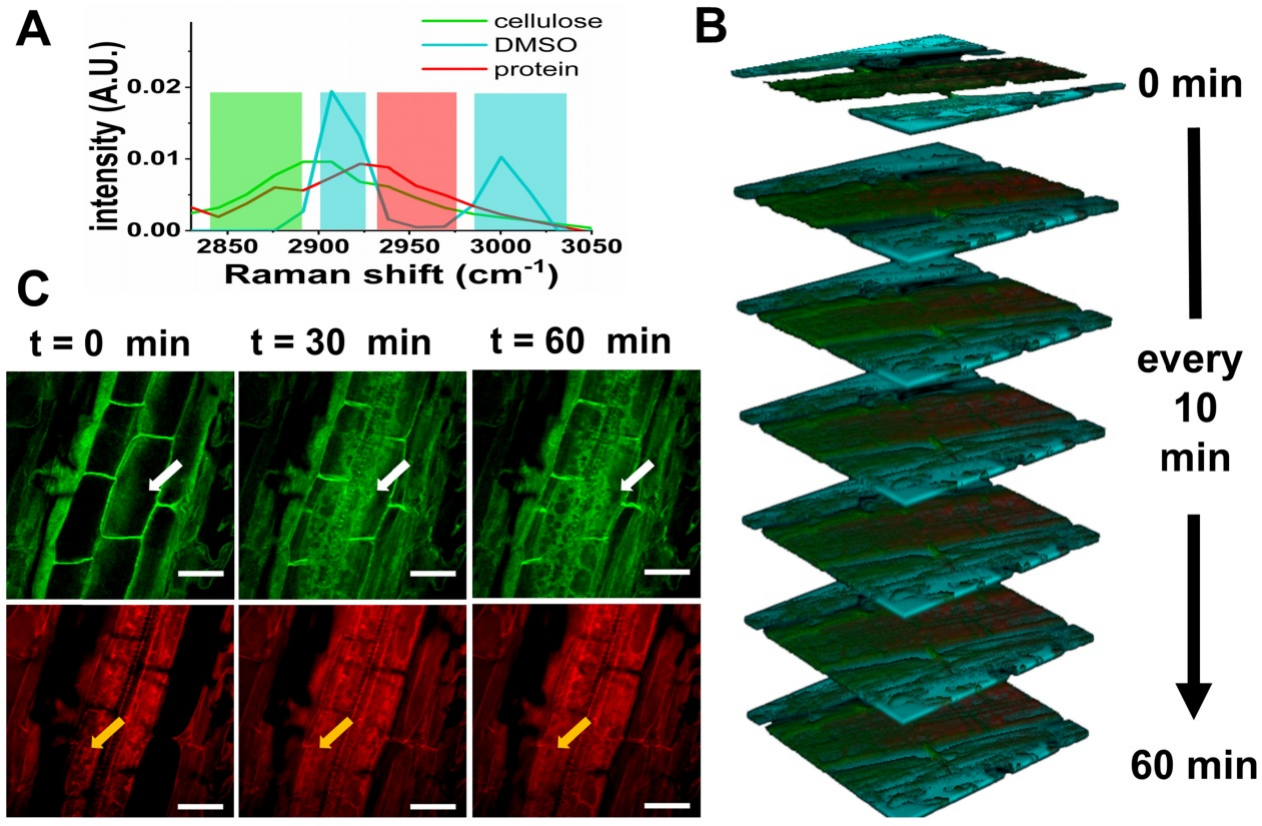
4D multiplexed SLM-SRS imaging of DMSO penetration into *Arabidopsis thaliana in vivo*. (A) SRS spectra of cellulose, DMSO and protein. The shaded areas represent sparse-sampling masks with colors matching to each spectrum. (B) 4D visualization of cellulose (green), DMSO (cyan) and protein (red) at a time interval of 10 min for 60 min (Supplementary Movie S4 at an actual interval of 5 min for 60 min). (C) 2D Snapshots of *Arabidopsis thaliana* at cellulose and protein channels. Over time, the deleterious effects of DMSO on the cell morphology and viability are visible (white and orange arrows). All images are obtained within 5 s with 2.4 µs of pixel dwell time for 256 x 256 pixels (100 x 100 µm) and averaging 9 times. 799 nm of the pump and 1041 nm of the Stokes beams are used. The average powers of the pump after passing through spectral filters for cellulose, protein and DMSO are 12, 15 and 16 mW respectively. 20 mW of the Stokes beam is used throughout the experiment. Time constant of lock-in amplifier is set at 2 µs. Scale bar = 20 µm.

To demonstrate *in vivo* SLM-SRS imaging, we have visualized the penetration of DMSO into *Arabidopsis thaliana*. DMSO is a common solvent used to deliver water-insoluble external agents into the plants, but its diffusivity and toxic effects on the plant tissue are poorly studied. We apply SLM-SRS technique to investigate the penetration rate as well as the acute effect of DMSO on the cellulose and proteins, which are the major components in plant tissue 20. We directly apply 5% of DMSO into a 3-day-old intact root of *Arabidopsis thaliana* and perform serial 3D SRS imaging with sparse-sampling masks alternated for multiplexed visualization of DMSO, cellulose and protein (Figure 8A). The entire 3D multiplexed image is acquired at a 5-min interval from 0 to 60 min (Figure 8B; for full movie, see Movie S4). In general, while the DMSO penetration into the outer epidermis layer is almost instant, further diffusion toward the core where phloem and xylem are located is found much slower. The decline in the diffusivity may be attributed to the increasing cell density toward the core 21. Consequently, the steady-state for the complete dissemination is only reached at 30 min. In the tissue architecture, the cellulose seems swollen by the DMSO uptake (green image in Figure 8C). Further, cellular damages due to the toxicity of DMSO is noticeable at the protein channel (red) possibly due to an increased accumulation of H_2_O_2_ 22, thus reflecting acute toxic effect of DMSO even at a low concentration.

## Discussion

By patterning the broadband excitation beam with the sparse-sampling mask that caters to the SRS spectrum of a specific molecule, we demonstrate that SRS sensitivity can be enhanced without compromise of chemical specificity. Initially, the sparse-sampling scheme was implemented in the spontaneous Raman micro-spectroscopy where its key advantage is to reduce image acquisition time due to the less number of mask-switching steps as compared to the sequential spectral scanning 11. The incorporation of the sparse-sampling method with SRS microscopy brings an additional benefit, apart from the chemical selectivity, that by shaping the pulse laser, it enables excitation of the sample with a higher peak power to generate greater SNR of SRS image. Such an improvement is independent of Felgett's signal-to-noise advantage 13 that marginally benefits from the shot-noise-limited detection level attained by our SRS imaging system. Our SLM-SRS work can be further distinguished from the spectrally tailored excitation-stimulated Raman scattering (STE-SRS) microscopy reported 23, in which the excitation mask is designed based on subtraction of desired signals from the interfering ones for maximizing specificity against the interfering components. As a result, the STE-SRS signal from the target component tends to be over-suppressed together with the interference signals. Such a compromised signal intensity may not be suitable for dynamic imaging for which a long integration time is hardly permitted.

The recent development of SRS spectroscopic technique based on resonant delay line and regularized non-negative matrix factorization algorithm can acquire a multiplexed image in 0.8 s 24. Our SLM-SRS technique shows superior imaging speed to afford the acquisition of chemical images within 0.2 s. However, such superiority is conditional for situations where Raman spectra of the target components in the sample are known for the mask design. To relax the requirement of the prior knowledge, our SLM offers dual function capabilities of the hyperspectral scanning and sparse-sampling excitation, whereby the former can be performed to obtain the necessary spectra prior to the latter. Thus, our SLM-SRS imaging technique developed is robust and versatile for rapid multiplexed vibrational imaging of any biological samples with high chemical sensitivity and specificity. We anticipate that by incorporating the spectra acquisition, MCR decomposition and sparse-sampling mask generation into a single algorithm, the 3-D SRS image processing time can be greatly reduced, thereby enabling real-time observation of unexplored biological events and *in vivo* biomedical systems.

In summary, we have developed a unique spatial light-modulated SRS (SLM-SRS) microscopy that spectrally shapes the broadband excitation beam to enhance the SNR without compromise of chemical selectivity. Our results show that SLM-SRS technique improves the SNR of PMMA and PS SRS images by 3- and 5-fold, respectively, compared to the narrowband excitation while retaining high chemical specificity. We also demonstrate that the pulse-shaping module based on the flexible SLM in the system can provide dual functions for spectral patterning and rapid acquisition of SRS spectra together, thus enabling our SLM-SRS technique to be independent of external instruments to gain prior knowledge of the unknown samples. We prove the capability of SLM-SRS microscopy for rapid 3-color vibrational imaging by visualizing the Brownian motion of the PS and PMMA beads in DMSO solutions at 70 ms intervals without any artifacts. Finally, we demonstrate the biological applicability of SLM-SRS microscopy by uncovering new biological insights associated with cell apoptosis and transport of toxic solute into the plant root *in vivo*. The current state-of-the-art of SRS microscopy is challenged by the increasing demand for dynamic imaging that requires higher sensitivity to afford faster imaging speed. Our SLM-SRS imaging technique developed is proven to be suitable for the rapid observations of dynamic processes in biological samples as well as *in vivo* biochemical imaging applications.

## Supplementary Material

Supplementary figures.

Movie S1.

Movie S2.

Movie S3.

Movie S4.

## Abbreviations

SLM-SRS: spatial light-modulated stimulated Raman scattering
SNR: signal-to-noise ratio
SLM: spatial light modulator
SRL: stimulated Raman loss
SRG: stimulated Raman gain
DMD: digital micromirror device
EOM: electro-optic modulator
MCR: multivariate curve resolution
PS: polystyrene
PMMA: poly methyl methacrylate
DMEM: Dulbecco's Modified Eagle Medium
DMSO: dimethyl sulfoxide
STE-SRS: spectrally tailored excitation-stimulated Raman scattering
