# Supplementary material for: Spatial light-modulated stimulated Raman scattering (SLM-SRS) microscopy for rapid multiplexed vibrational imaging
Source: Theranostics. 2020 Jan 1;10(1):312–22. doi: 10.7150/thno.38551 (PMC6929623; doi:10.7150/thno.38551)

Spatial light-modulated stimulated Raman scattering (SLM-SRS) microscopy for rapid multiplexed vibrational imaging

KIDEOG BAE[1], WEI ZHENG[1], ZHIWEI HUANG[1]*

[1]*Optical Bioimaging Laboratory, Department of Biomedical Engineering, Faculty of Engineering, National University of Singapore, Singapore 117576*

***Correspondence to**:
Dr. Zhiwei Huang, Optical Bioimaging Laboratory, Department of Biomedical Engineering, Faculty of Engineering, National University of Singapore, 9 Engineering Drive 1, Singapore 117576
Tel: +65- 6516-8856
Fax: +65- 6872-3069
E-mail: biehzw@nus.edu.sg

Running title: SLM-SRS microscopy for rapid multiplexed vibrational imaging

Generation of sparse-sampling masks for PS, PMMA and DMSO

For demonstration of the rapid multiplexed ability of SLM-SRS, we first validate SLM-SRS imaging specificity of 4.5 μm polystyrene (PS) and 3.0 μm poly (methyl methacrylate) (PMMA) and DMSO. The sparse-sampling masks for the three chemicals are generated based on their corresponding SRS spectra (Figure S2A). Figure S2B-D shows the SLM-SRS images of DMSO, PS and PMMA beads. The beads are imaged separately from the DMSO solution to avoid Brownian motion.

Generation of sparse-sampling masks for cellular lipid and protein

Prior to the 4D multiplexed SLM-SRS imaging of live HeLa cells, we generate sparse-sampling masks specific to the major components in the cells. We first obtain the SRS spectrum of the cells by hyperspectral scanning from 2830-3000 cm^{-1}. The spectra are then decomposed using multivariate curve resolution-alternative least square (MCR-ALS) algorithm [1]. The number of the major components determined by principal component analysis (PCA) is two (Figure S3). Subsequently, the spectra identified are fed into the sparse-sampling algorithm to generate the corresponding masks (shaded area in Figure S3 represents the 'pass' region).

Supplementary Figure Captions

Figure S1. Measurement of the spatial resolutions of SLM-SRS microscopy. (A) SLM-SRS image of 0.3 μm PMMA beads. Line profiles (orange arrow) of the bead along (B) x-y, and across (C) z. FWHMs of the lateral and axial resolutions are estimated to be 0.45 and 2.1 μm, respectively. Scale bar = 0.5 μm.

Figure S2. Validation of SLM-SRS for poly (methyl methacrylate) (PMMA) and polystyrene (PS) beads and dimethyl sulfoxide (DMSO). (A) SRS spectra of PS (green), PMMA (red) and DMSO (cyan). The shaded areas represent sparse-sampling masks with colors matching to each SRS spectrum. The SLM-SRS images of (B) DMSO, (C) PS and (D) PMMA beads. (E) Overlay image of (C) and (D). Scale bar = 10 μm.

Figure S3. MCR spectra obtained from the SRS spectrum of live HeLa cells. Two spectra of cellular lipid (green) and cellular protein (magenta) are generated. The shaded areas represent sparse-sampling mask with colors matching to each MCR spectra.

Supplementary Movies Captions

Movie S1. Multiplexed SLM-SRS image stacks of PS and PMMA under Brownian motion in DMSO. The three-color images are acquired at intervals of 200 ms with 0.8 μs of pixel dwell time for the 40 × 40 μm (96 × 96 pixels). 797 nm of the pump and 1041 nm of the Stokes beams are used. The average powers of the pump after passing through spectral filters for PS, PMMA and DMSO are 10, 16 and 18 mW, respectively. Time constant of lock-in amplifier is set as 0.8 μs. Scale bar = 5 μm.

Movie S2. Multiplexed SLM-SRS image stacks of live Hela cells in the alkaline environment at pH of 8.5. Scale bar = 7 μm. All images are obtained within 40 s with 2.4 μs of pixel dwell time for 320 x 320 pixels (80 x 80 μm) and averaging 9 times. 799 nm of the pump and 1041 nm of the Stokes beams are used. The average powers of the pump after passing through sparse-sampling mask for cellular lipid and protein are 20 and 23 mW, respectively. 20 mW of the Stokes beam is used throughout the experiment. Time constant of lock-in amplifier is set as 2 μs. Scale bar = 7 μm.

Movie S3. Multiplexed SLM-SRS image stacks of live HeLa cell in PBS media (pH 7.4) as control experiment for Fig. 5. Scale bar = 7 μm. All images are obtained within 20 s with 2.4 μs of pixel dwell time for 160 x 160 pixels (40 x 40 μm) and averaging 9 times. 799 nm of the pump and 1041 nm of the Stokes beams are used. The average powers of the pump after passing through sparse-sampling mask for cellular lipid and protein are 20 and 23 mW, respectively. 20 mW of the Stokes beam is used throughout the experiment.

Movie S4. 4D multiplexed SLM-SRS image stacks of DMSO penetration into 3D *Arabidopsis thaliana* root. All images are obtained within 5 s with 2.4 μs of pixel

dwell time for 256 x 256 pixels (100 x 100 μm) and averaging 9 times. 799 nm of the pump and 1041 nm of the Stokes beams are used. The average powers of the pump after passing through spectral filters for cellulose, protein and DMSO are 12, 15 and 16 mW, respectively. 20 mW of the Stokes beam is used throughout the experiment. Time constant of lock-in amplifier is set at 2 μs. Scale bar = 20 μm.

Reference

1. Ruckebusch C, Blanchet L. Multivariate curve resolution: A review of advanced and tailored applications and challenges. Anal Chim Acta. 2013; 765: 28-36.

Figure S1

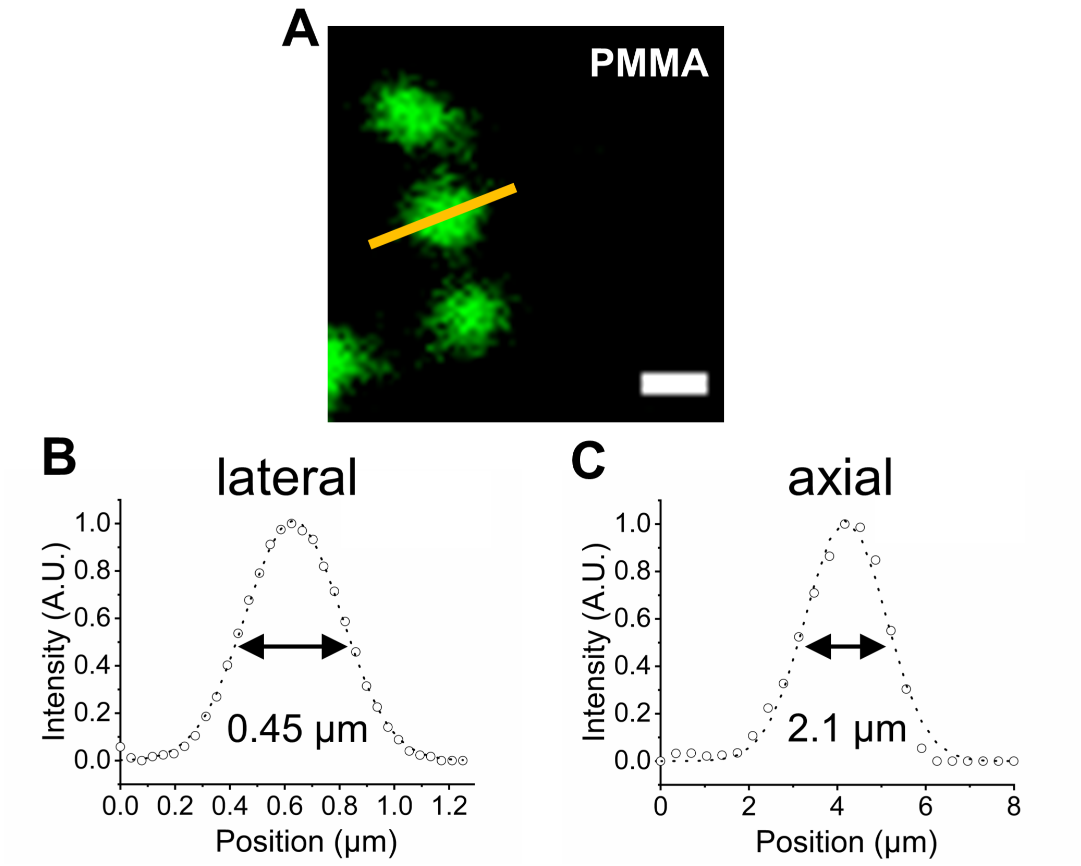
A
PMMA
B
lateral
Intensity (A.U.)
1.0
0.8
0.6
0.4
0.2
0.0
0.45 μm
0.0 0.2 0.4 0.6 0.8 1.0 1.2
Position (μm)
C
axial
Intensity (A.U.)
1.0
0.8
0.6
0.4
0.2
0.0
2.1 μm
0 2 4 6 8
Position (μm)

Figure S2

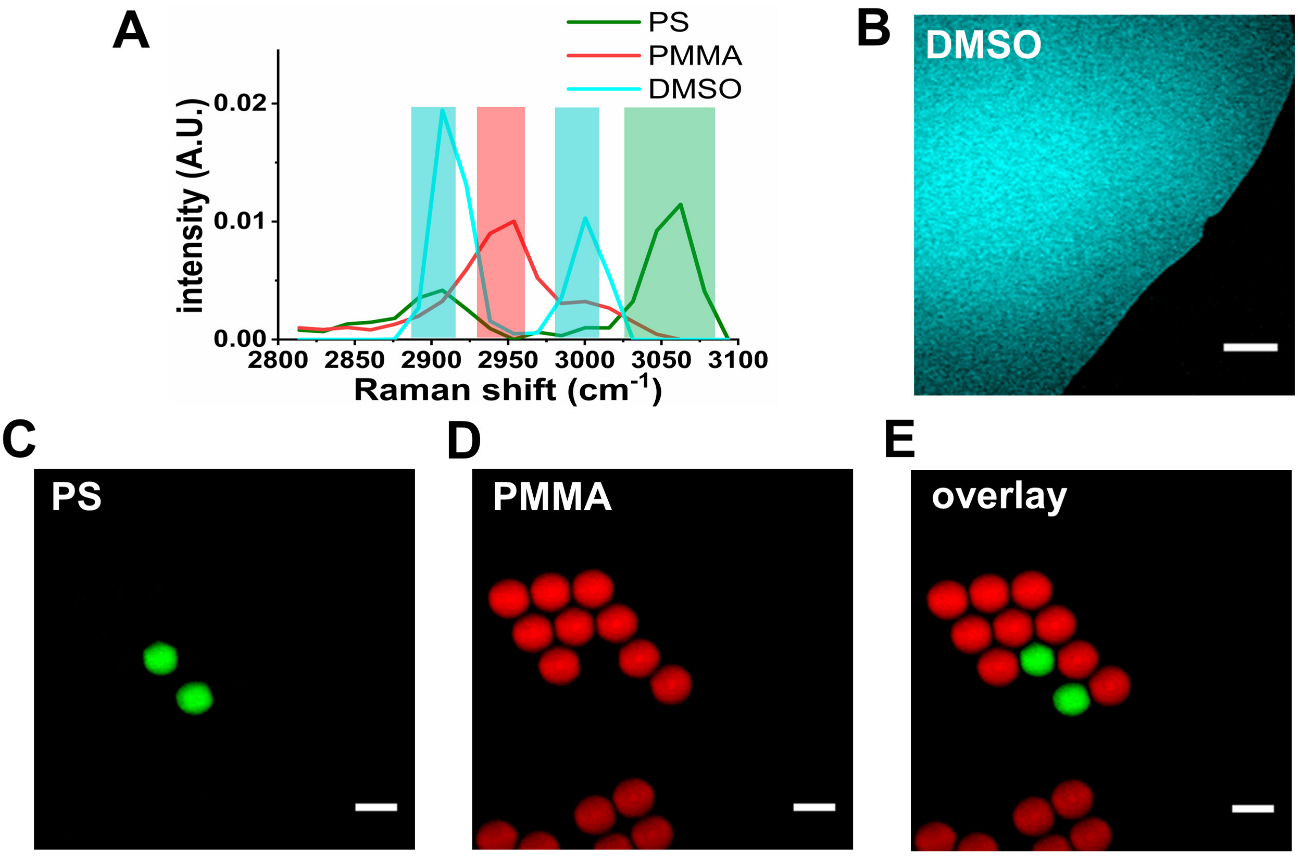

A
PS
PMMA
DMSO
intensity (A.U.)
0.02
0.01
0.00
2800
2850
2900
2950
3000
3050
3100
Raman shift (cm^{-1})
B
DMSO
C
PS
D
PMMA
E
overlay

Figure S3

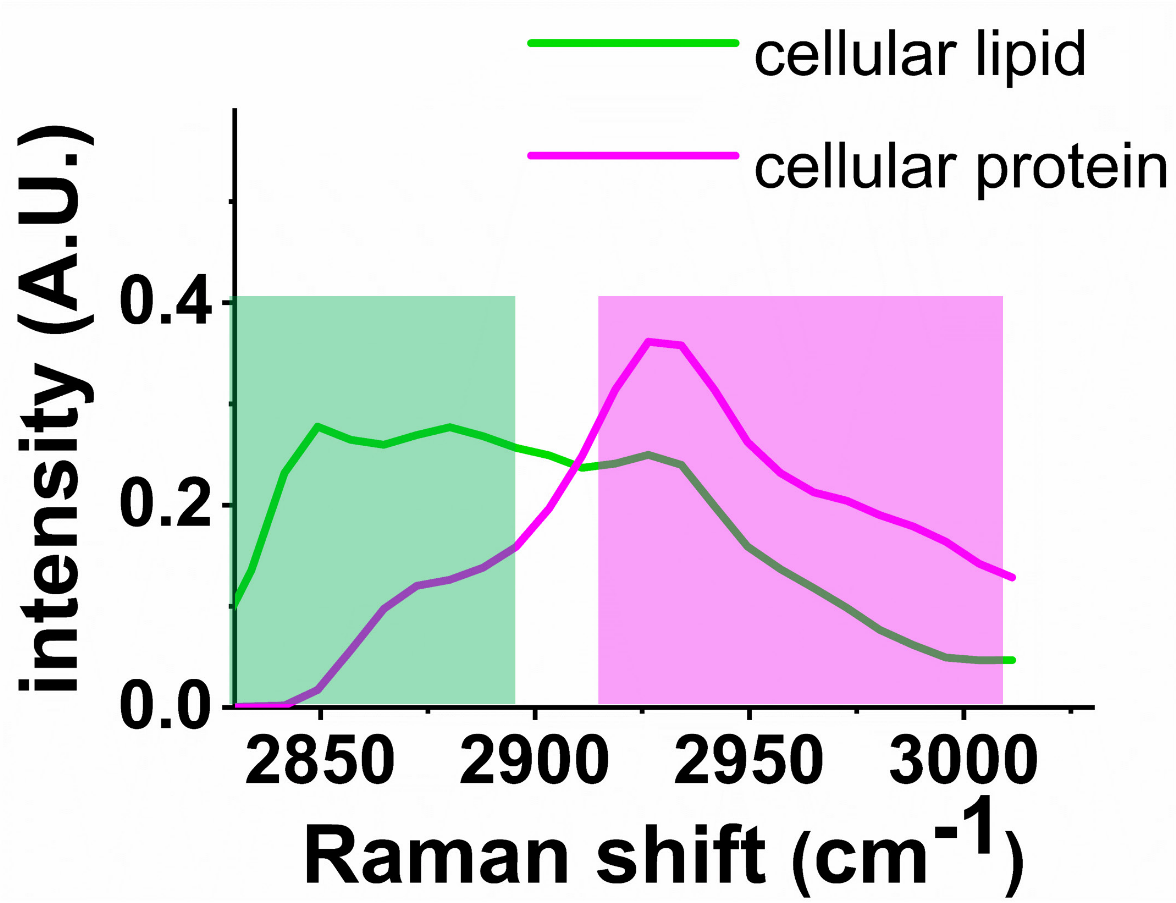
cellular lipid
cellular protein
intensity (A.U.)
0.0
0.2
0.4
2850
2900
2950
3000
Raman shift (cm^{-1})

Movie S1

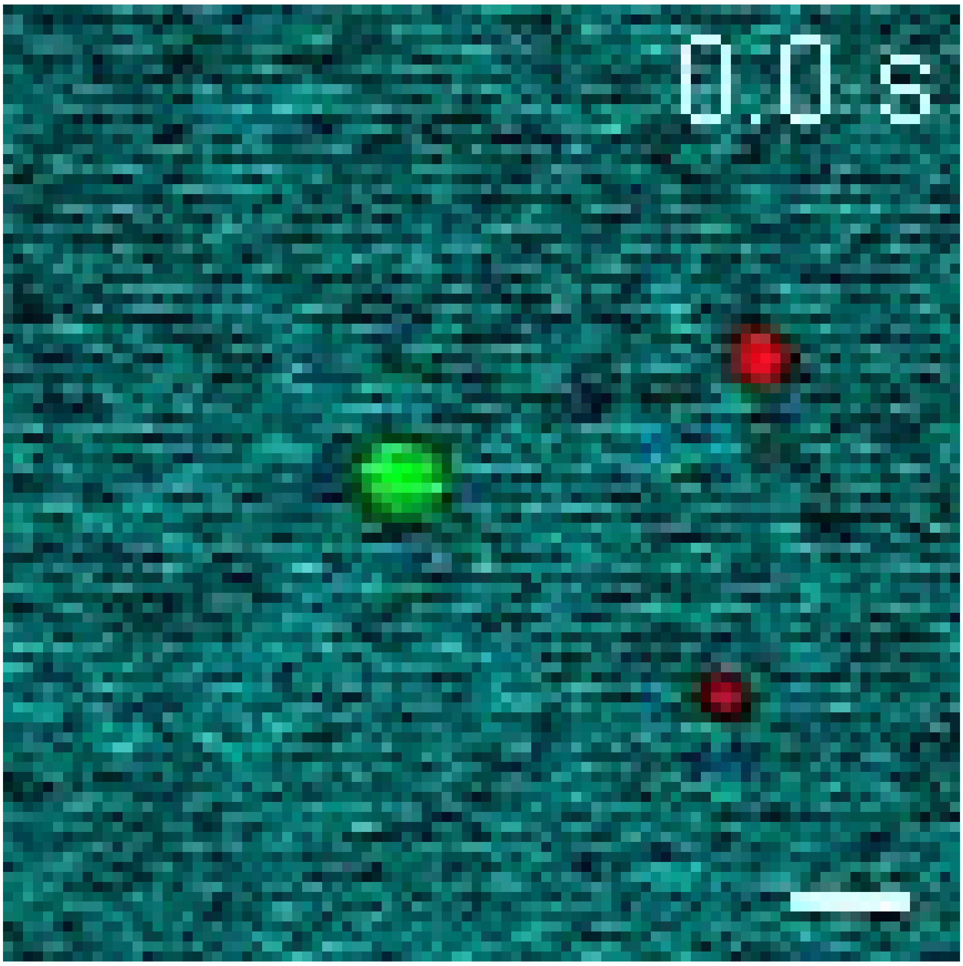

Movie S2

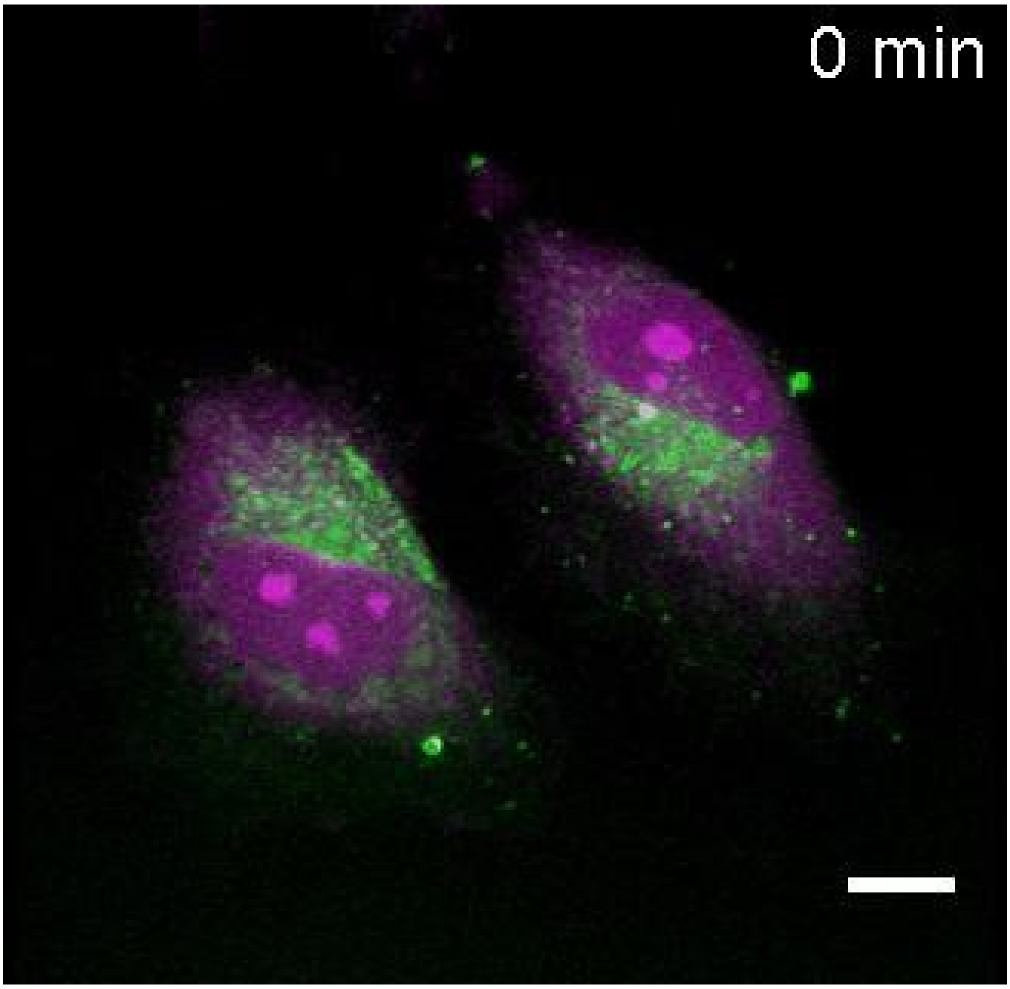

Movie S3

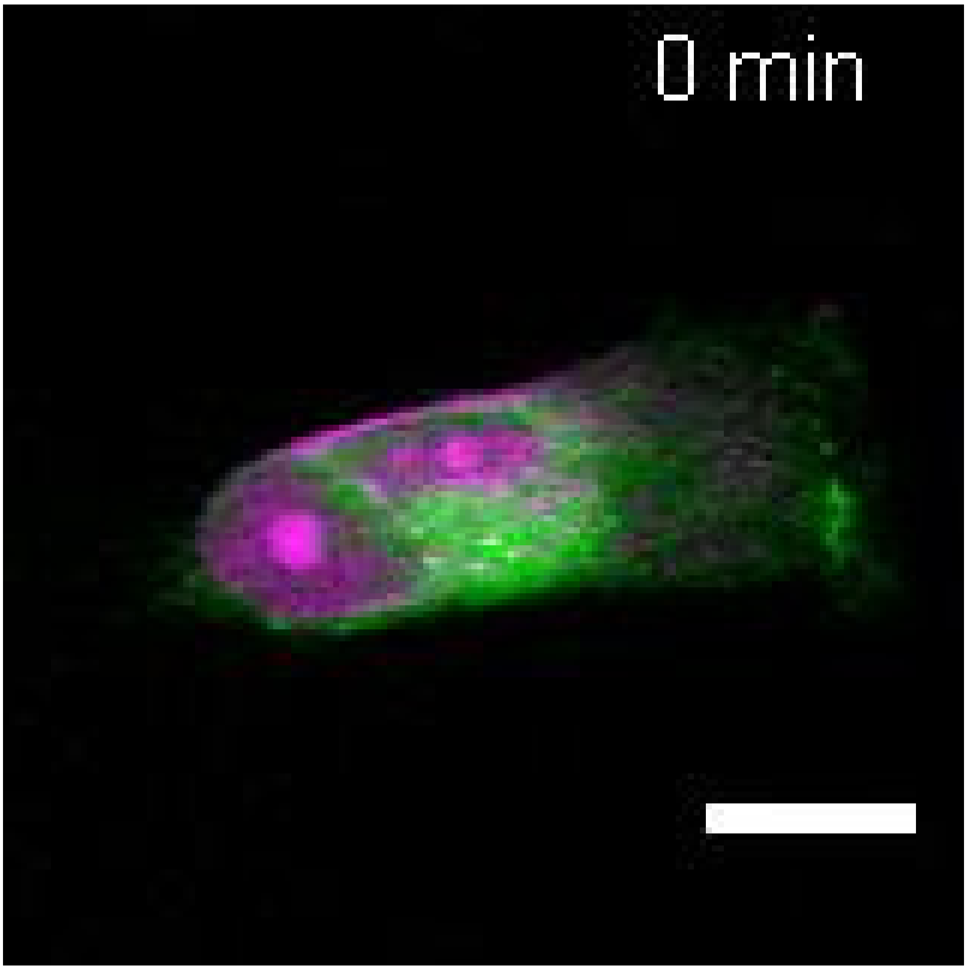

Movie S4

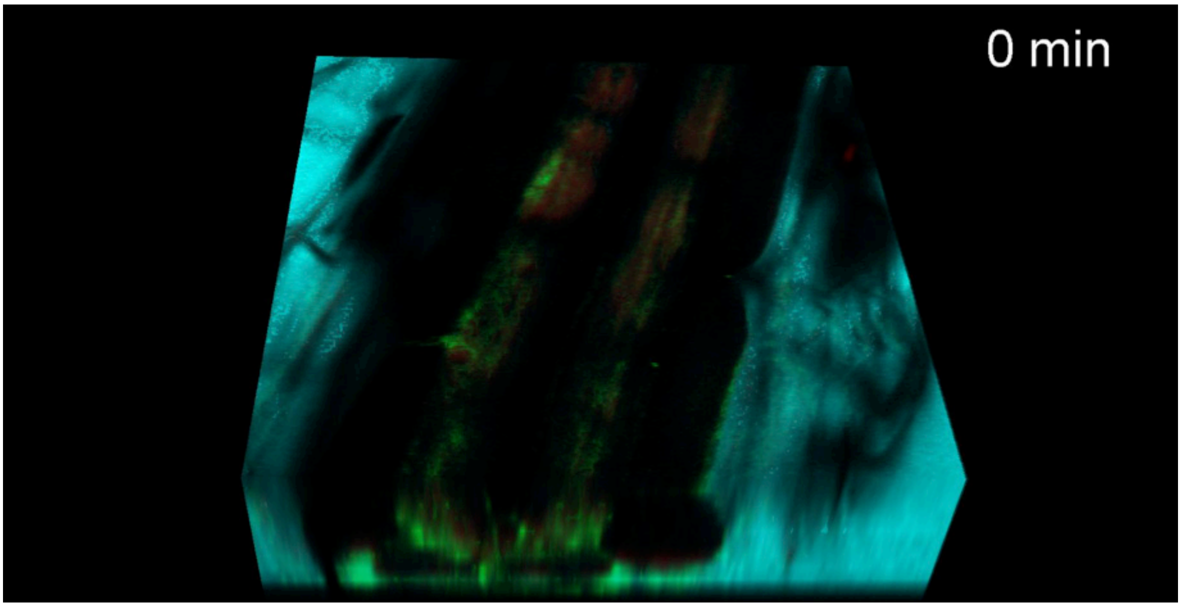

Supplement: Supplementary file 1 — Supplementary figures. [file thnov10p0312s1.pdf]
